# Supplementary material for: Large-scale microbiome data integration enables robust biomarker identification
Source: Nat Comput Sci. 2022 May 23;2(5):307–16. doi: 10.1038/s43588-022-00247-8 (PMC10766547; doi:10.1038/s43588-022-00247-8)
Supplement: Supplementary file 1 — Supplementary Figs. 1–8 and Tables 1 and 2. [file 43588_2022_247_MOESM1_ESM.pdf]

nature portfolio

https://doi.org/10.1038/s43588-022-00247-8

Supplementary information

Large-scale microbiome data integration enables robust biomarker identification

In the format provided by the authors and unedited

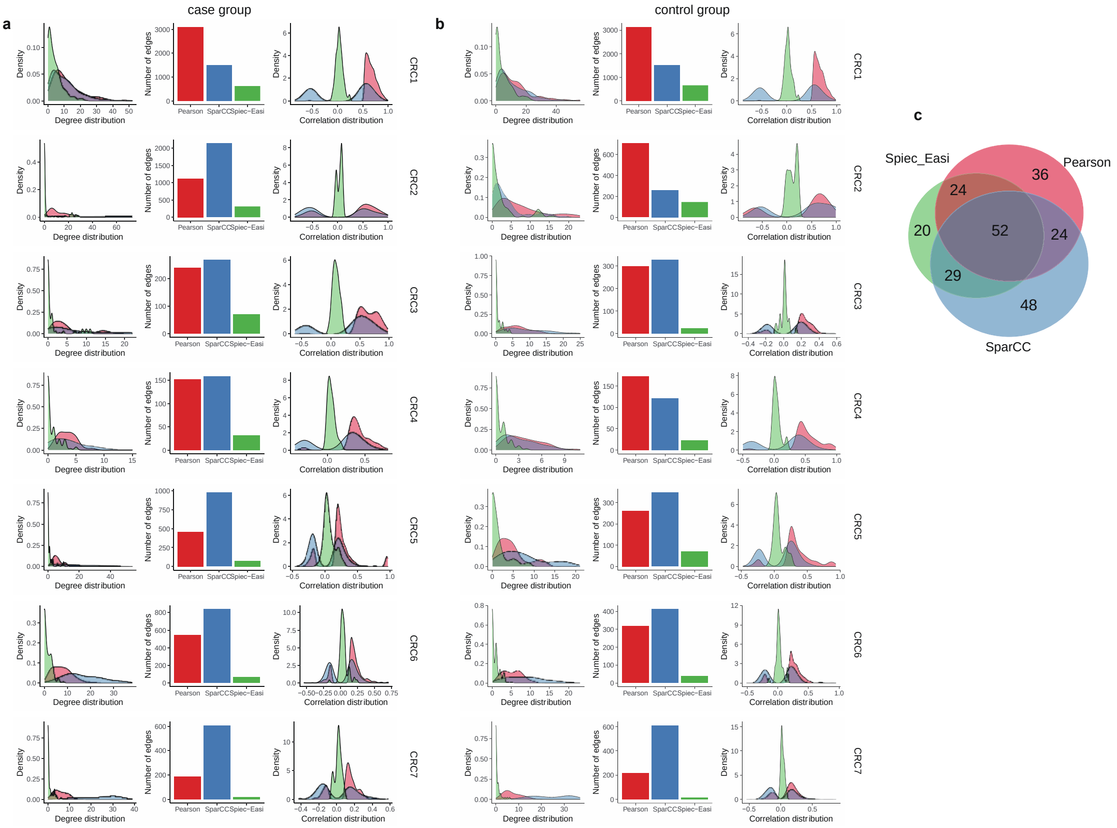

Supplementary Figure 1. Comparison of different network construction methods. a, Topological parameters of seven CRC microbial networks in the case group (P < 0.01). b, Topological parameters of seven CRC microbial networks in the control group (P < 0.01). c, The overlap of markers identified by NetMoss based on different network construction methods.

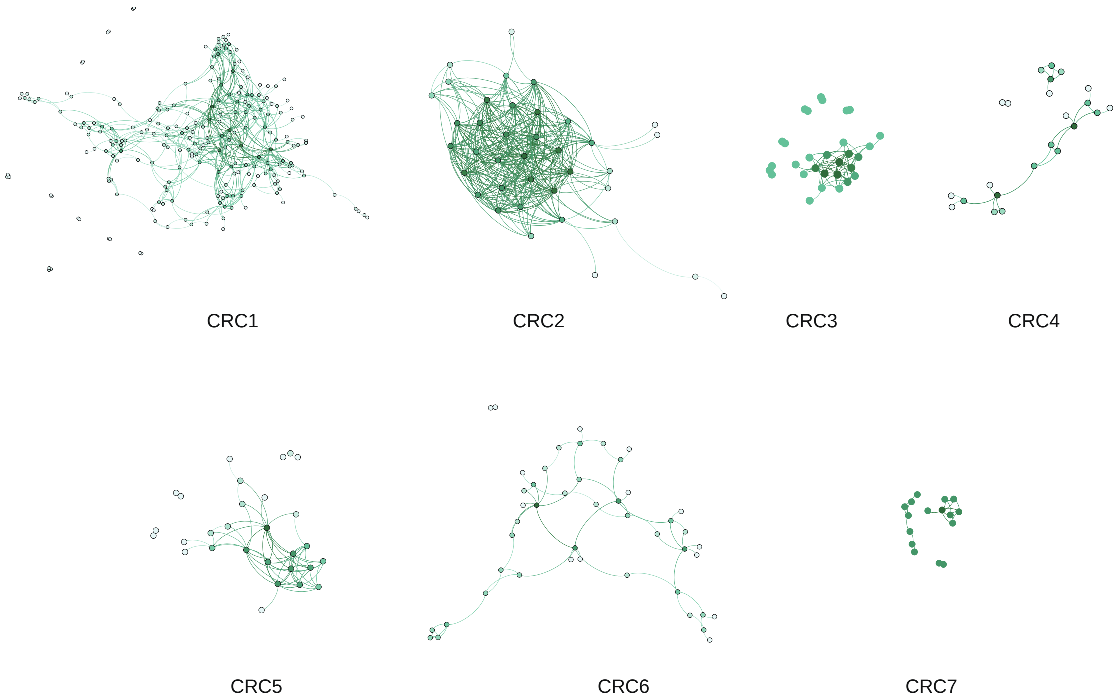

Supplementary Figure 2. Co-occurrence networks constructed from study CRC 1 – CRC 7. Different colors of dots in the networks represent different network degree.

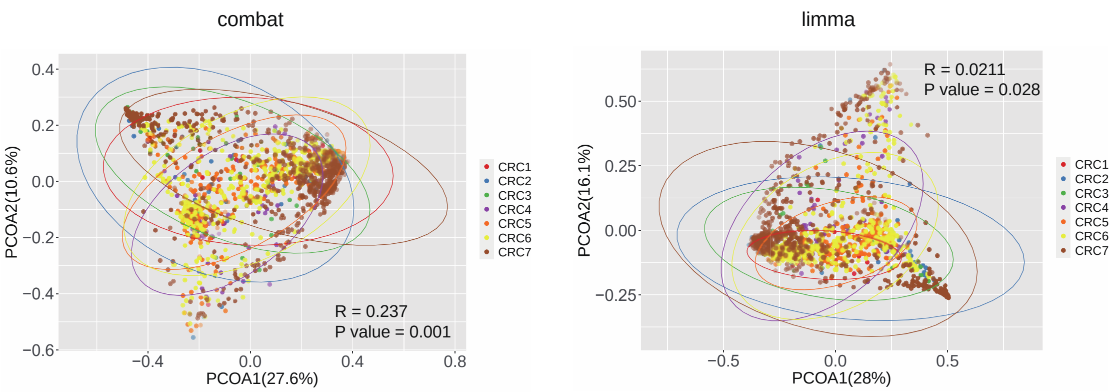

Supplementary Figure 3. Batch effects of seven datasets based on abundance integration. Datasets were processed by combat (left) and limma (right), respectively. The color of nodes represents different studies. The circles refer to 95% confidence intervals.

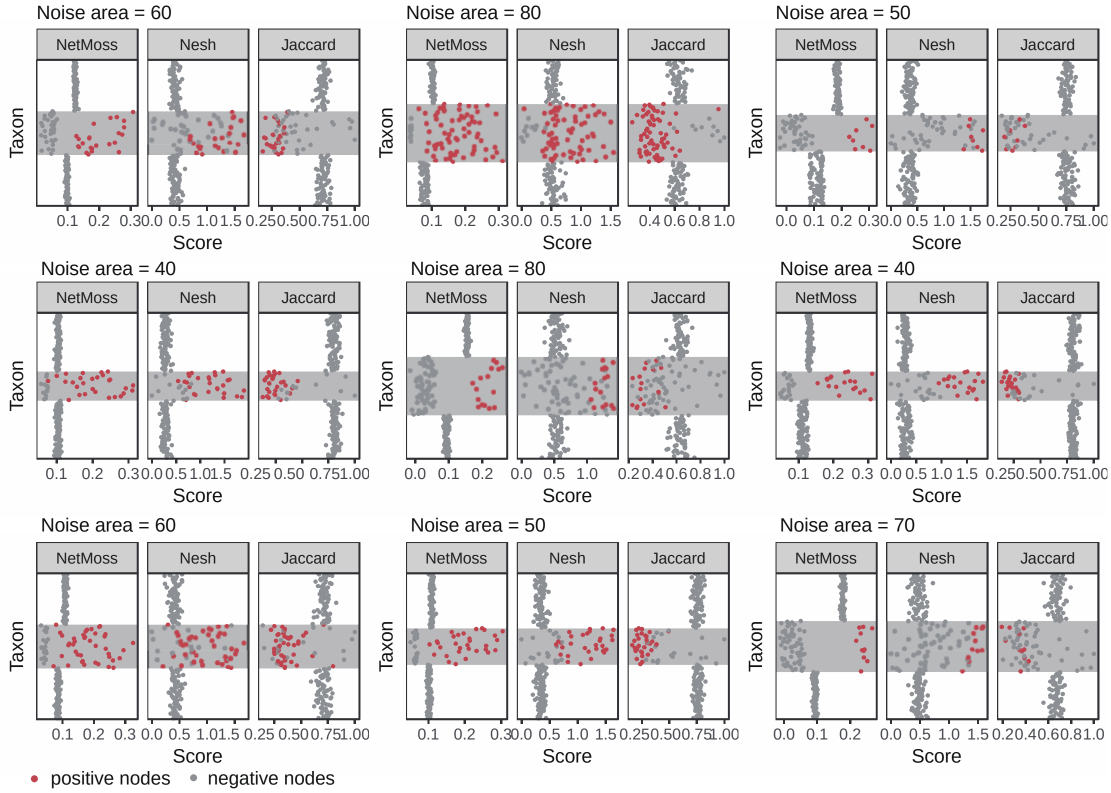

Supplementary Figure 4. Score distribution of different methods under various noise levels. Datasets were evaluated by NetMoss score (left), Neighbor shift score (middle), and Jaccard edge index (right), respectively. Red nodes correspond to transited submodules and gray nodes correspond to other submodules. The gray areas in the boxes represent noise areas under different noises.

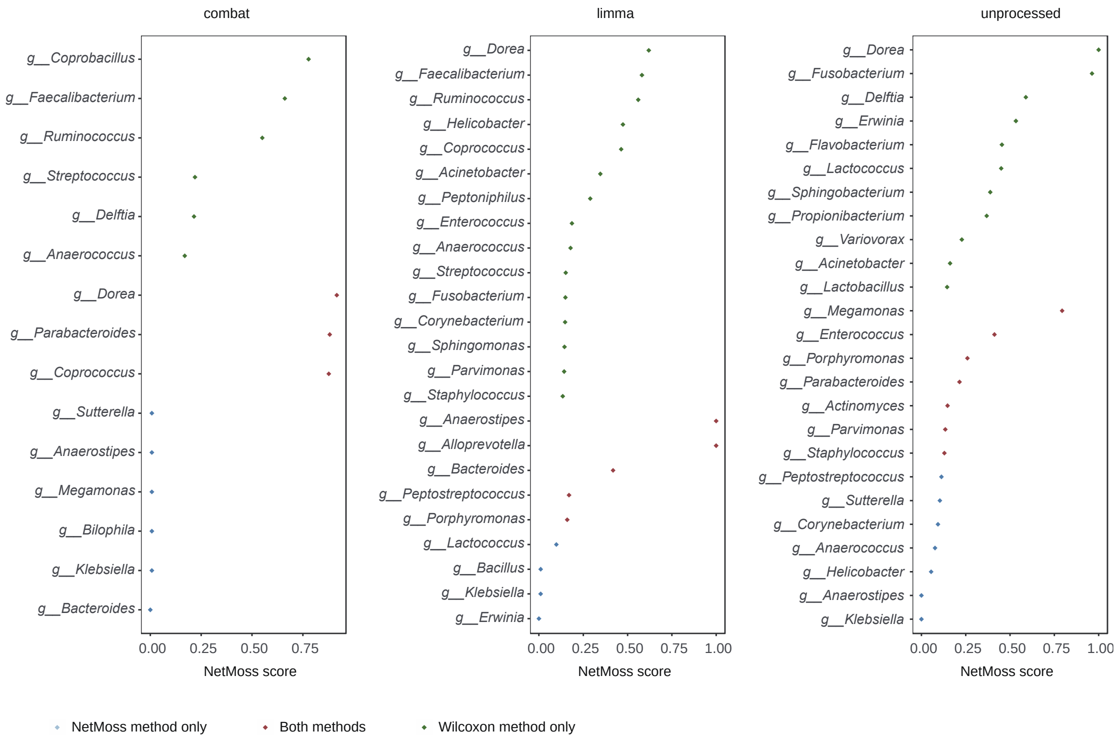

Supplementary Figure 5. NetMoss score of different processed groups. Integrated based on abundance and processed by combat (left), limma (middle), or Wilcoxon (right).

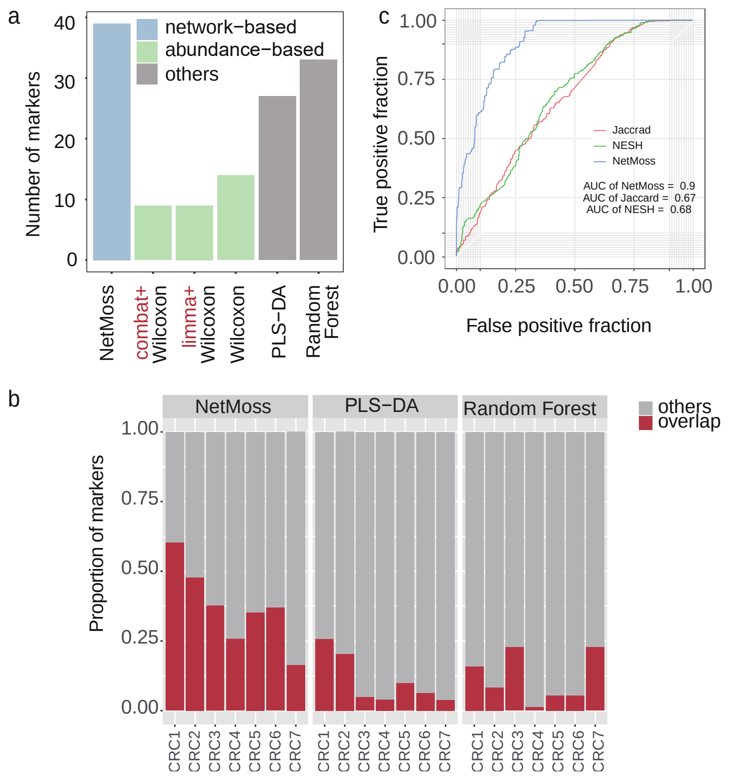

Supplementary Figure 6. Classification comparison of different methods in seven CRC studies. a, Number of markers identified by different methods. b, Overlap between markers identified by each study individually and by the combined datasets. c, Prediction power of three network-based methods.

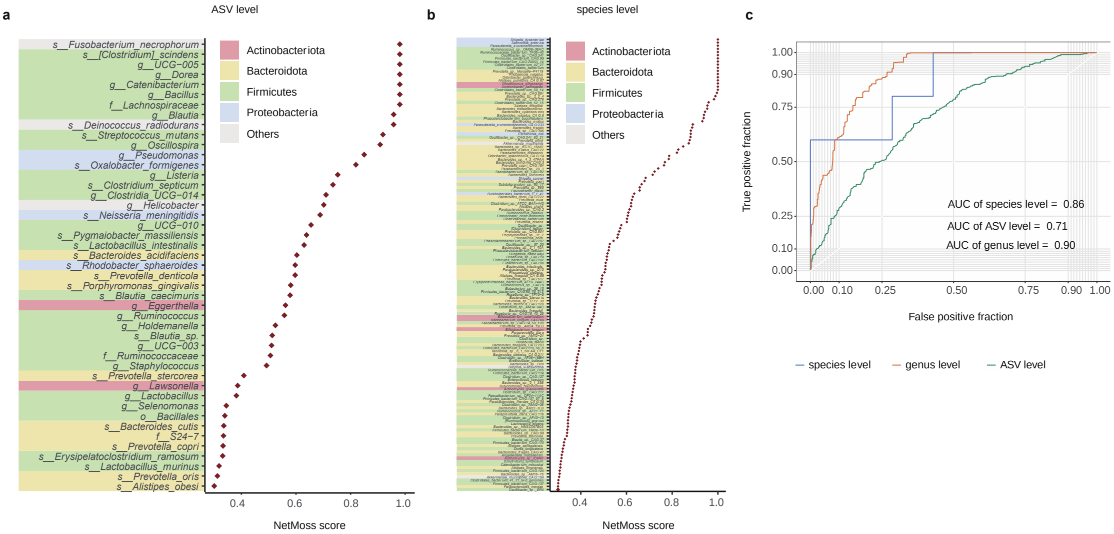

Supplementary Figure 7. Marker identification at different taxonomic levels. a, Markers identified by NetMoss at the ASV level. b, markers identified by NetMoss at the species level. c, Prediction power at three different taxonomic levels (genus level and ASV level from 16S dataset, and species level from metagenome dataset).

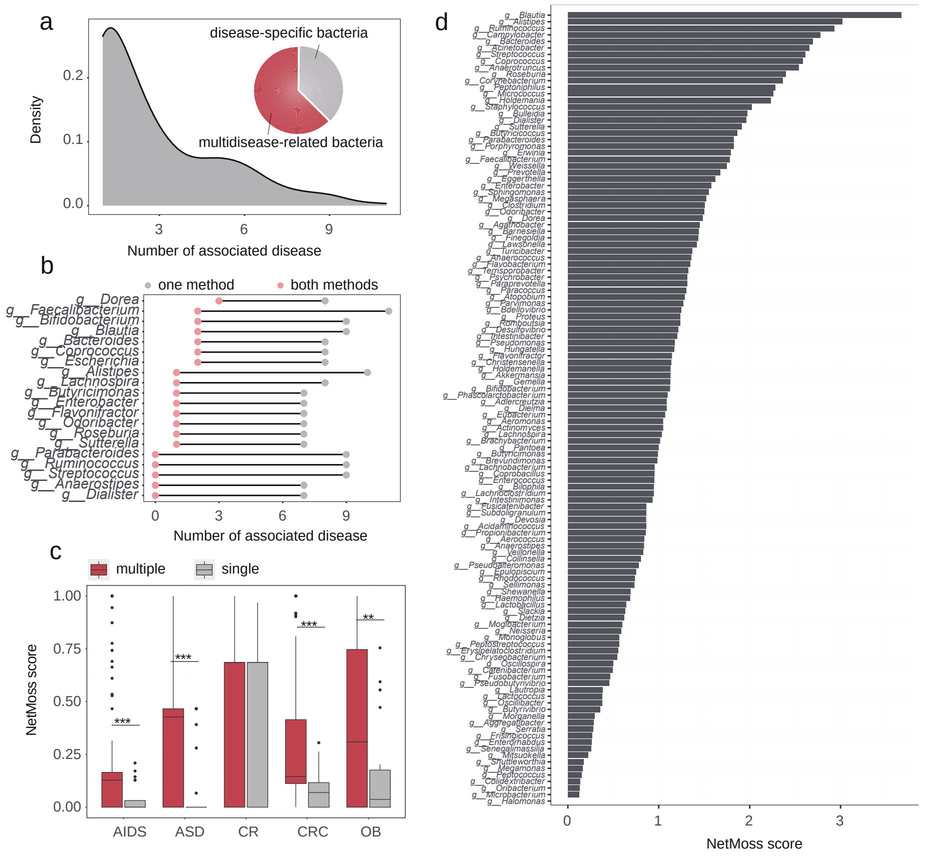

Supplementary Figure 8. Significant bacteria associated with multiple diseases. a, The distribution of significant genera associated with various diseases. The small pie plot refers to proportion of disease-specific bacteria and multidisease-related bacteria in the analysis of 12 diseases. b, Top 20 bacteria which are significantly associated with multiple diseases. Pink dots represent significant genera identified by both the abundance-based method and NetMoss. Gray dots represent significant genera identified either by the abundance-based method or NetMoss. c, NetMoss scores of multidisease-related bacteria (red) and disease-specific bacteria (gray) in five diseases. The upper and lower horizontal lines of the box represent the 25th and 75th percentiles respectively, the horizontal midline represents the median, the upper and lower horizontal whiskers lines are the upper and lower limits and the dots are the outliers. Two-sided Wilcoxon test. ***, P < 0.001; **, P < 0.01; *, P < 0.05. d, NetMoss score of multidisease-related bacteria in the combined network.

Supplementary Table 1. The microbiota datasets used in this study

Dataset	Accession	#Controls	#Cases	Platform	Country	16S Region	Sample Type
AC	PRJNA449243	31	31	HiSeq	China	V4	severe pustular acne vulgaris
AITD	PRJNA450230	28	74	HiSeq	China	V3-V4	autoimmune thyroid
AP	PRJNA214550	10	26	454	USA	V1-V2	appendix and rectal samples
A-P	PRJNA428226	100	148	MiSeq	USA	V4	aspirin
ART	PRJNA203810	28	86	454	USA	V1-V2	arthritis
ASD1	PRJNA355023	24	30	NextSeq	India	V3	autism spectrum disorder
ASD2	PRJNA282013	44	59	MiSeq	USA	V1-V2	autism spectrum disorder
ASD3	PRJNA327785	0	40	MiSeq	China	V3-V4	autism spectrum disorder
CAP	PRJNA294937	0	48	MiSeq	China	V3-V4	community-acquired pneumonia
CDI	PRJNA270936	0	25	454	China	V3-V4	clostridium difficile infection
CDI1	PRJNA274722	56	60	MiSeq	China	V3	clostridium difficile infection
CDI2	PRJNA307992	86	144	MiSeq	USA	V4	clostridium difficile infection
CHB	PRJNA382861	57	206	MiSeq	China	V3-V4	chronic Hepatitis B
CR1	PRJNA298762	20	10	MiSeq	Canada	V3	Crohn’s disease
CR2	PRJNA257186	36	21	454	USA	V1-V2	Crohn’s disease
CR3	PRJNA428898	9	26	MiSeq	China	V4-V5	Crohn’s disease
CRC1	PRJNA288419	23	23	454	China	V3	colorectal cancer
CRC2	PRJNA269561	23	35	HiSeq	China	V3	colorectal cancer
CRC3	PRJNA430990	247	36	MiSeq	China	V3-V4	colorectal cancer
CRC4	PRJEB6070	50	79	HiSeq	Germany	V4	colorectal cancer
CRC5	PRJNA318004	141	263	MiSeq	USA	V4	colorectal cancer
CRC6	PRJNA290926	190	352	454	USA	V4	colorectal cancer
CRC7	PRJNA445346	613	667	MiSeq	USA	V3-V6	colorectal cancer
CS1	PRJNA383300	0	94	MiSeq	China	V3-V4	constipation
CS2	PRJNA401944	103	54	MiSeq	China	V4-V5	constipation
DI	PRJNA275256	0	60	MiSeq	China	V3-V4	diarrhea
DI1	PRJNA416445	28	52	MiSeq	Spain	V4	diarrhea
Dp	PRJNA251678	0	5	454	China	V1-V3	depression
E	PRJNA318088	21	11	MiSeq	China	V4-V5	environment
EN	PRJNA379186	30	144	MiSeq	China	V4	E. vermicularis infection
EP	PRJNA362482	30	28	MiSeq	China	V4-V5	refractory epilepsy

GDM	PRJNA418057	93	93	MiSeq	China	V3-V4	gestational diabetes mellitus
GDM1	PRJNA438491	0	82	MiSeq	Italy	V3-V4	gestational diabetes mellitus
GT	PRJNA359624	26	26	MiSeq	China	V3-V4	gout
H1	PRJNA388732	104	0	MiSeq	China	V4	health
H10	PRJNA418394	20	0	MiSeq	China	V3-V4	health
H11	PRJEB23227	24	0	MiSeq	China	V4-V5	health
H12	PRJNA388322	50	0	MiSeq	China	V1-V2	health
H13	PRJDB4360	469	0	MiSeq	Japan	V3-V4	health
H2	SRP012940	77	0	454	China	V1-V2	health
H3	PRJNA386614	65	0	MiSeq	China	V3-V4	health
H4	PRJNA314010	36	0	454	China	V1-V2	health
H5	PRJNA385551	1352	0	MiSeq	China	V3-V4	health
H6	PRJNA349463	120	0	MiSeq	China	V4	health
H7	PRJNA234071	35	0	454	China	V1-V3	health
H8	PRJNA414683	134	0	MiSeq	USA	V3-V4	health
H9	PRJNA324452	168	0	MiSeq	China	V4-V5	health
HCC	PRJEB8708	0	15	MiSeq	China	V3-V4	hepatocellular carcinoma
HCC1	PRJNA445357	0	26	MiSeq	Japan	V3-V4	hepatocellular carcinoma
HIV	PRJNA328008	0	62	MiSeq	France	V3	human immunodeficiency virus
HIV1	PRJNA233597	15	21	454	USA	V3-V5	human immunodeficiency virus
HIV2	PRJEB4335	13	23	MiSeq	USA	V4	human immunodeficiency virus
HIV3	PRJNA307231	34	205	MiSeq	Spain	V3-V4	human immunodeficiency virus
IBD	PRJNA431126	38	286	MiSeq	China	V4	inflammatory bowel disease
K	PRJNA382644	13	13	HiSeq	China	V3-V4	kidney stones
LC	PRJNA445763	20	36	MiSeq	China	V3-V4	liver cirrhosis
LE1	PRJNA451154	0	116	MiSeq	USA	V4	acute lymphoblastic leukemia
LE2	PRJNA449103	0	406	MiSeq	USA	V1-V3	acute lymphoblastic leukemia
M	PRJNA388136	20	20	HiSeq	China	V4	half-marathon runners
MD	PRJNA278793	0	114	MiSeq	China	V4	mental disorders
MHE	PRJNA174838	26	51	454	China	V1-V2	human monocytic ehrlichiosis

NASH	PRJDB2229	23	32	454	China	V1-V2	Non-alcoholic steatohepatitis
OB1	PRJNA486071	39	38	MiSeq	China	V4	obesity
OB2	PRJNA401981	40	40	MiSeq	USA	V3-V4	obesity
OB3	PRJNA433269	76	96	PGM	Mexico	V3	obesity
OB4	PRJNA339739	78	112	PGM	Mexico	V3	obesity
OS	PRJNA359375	6	12	MiSeq	China	V3-V4	Prader-Willi syndrome
PCOS	PRJNA341567	15	33	MiSeq	China	V3-V4	polycystic ovary syndrome
PD1	PRJEB4927	74	74	454	Finland	V1-V3	Parkinsons disease
PR	PRJNA360073	201	255	PGM	Denmark	V3	probiotics
SBS1	PRJNA434046	0	66	MiSeq	China	V1	short bowel syndrome
SBS2	PRJDB4453	3	21	MiSeq	Japan	V3-V4	short bowel syndrome
SBS3	PRJNA275923	7	11	MiSeq	Sweden	V2-V4	short bowel syndrome
SBS4	PRJNA309478	5	28	MiSeq	China	V4-V5	short bowel syndrome
SBS5	PRJDB4453	0	24	MiSeq	Japan	V3-V4	short bowel syndrome
T2D	PRJNA217953	144	144	454	China	V3	type 2 diabetes
TC	PRJNA477766	40	30	HiSeq	China	V3-V4	thyroid carcinoma
W	PRJEB12320	38	60	MiSeq	China	V3-V4	Whipple surgery
CRC	PRJNA763023	20	20	NovaSeq	China	metagenome	colorectal cancer

Supplementary Table 2. AUC of simulated datasets under different noise levels.

Permutation	NetMoss	NESH	JEI
1	0.91	0.86	0.86
2	0.98	0.85	0.85
3	0.93	0.85	0.79
4	0.94	0.68	0.48
5	0.92	0.86	0.86
6	0.99	0.92	0.83
7	0.98	0.92	0.89
8	0.96	0.91	0.59
9	0.99	0.94	0.86
10	0.95	0.96	0.70
